# Some Syphilinum Cases

**Published:** 1896-05

**Authors:** H. C. Morrow

**Affiliations:** Austin, Texas


### SOME SYPHILINUM CASES.

H. C. Morrow, Austin, Texas.

(Continued from March Number.)

### Case VI.

Mrs. Mc., age, sixty-five. Eczema on legs. Itches intensely at night. Has to sit up in chair until toward morning. Itches worse when she undresses and when the legs hang down. Always worse in the evening after the sun goes down and at night. Worse getting warm and bathing.

Eruption when first comes out moist, profuse discharge of serum from surface; afterward dry and scaly. Urine nearly as thick as castor oil, yellow, strong. Feels good to scratch, but scratching aggravates. Burning and itching worse before a rain. Itching worse around ankles and tops of feet. Bathing with cold water relieves, warm aggravates.

Syphilinum$^{cm}$ (Swan) gave her a great deal of relief and comfortable nights. I believe it would have cured had she kept up the treatment, but she removed to another State and I lost trace of her.

### Case VII.

Mr. F., age, fifty-two. Very stout, face fat and bluish. About five years since a warty-looking tumor came beneath left ear on the side of the neck. Had a "famous" New York specialist remove it with a salve.

A few months after scar commenced to enlarge rapidly, became hard, blue, and painful to touch, showing a return of the cancerous condition.

Had frequent sharp, shooting pains in the tumor, and it itched and burned.

Not desiring to go through the same torture again, he applied to me for relief.

I put him on Syphilinum$^{cmm}$ (Swan), and in a short time improvement became very manifest. The pains ceased, the blue color left, and instead of increasing it commenced to decrease, and in a few months it presented the appearance of a perfectly healthy normal scar, and has so remained until this day.

